# Measuring effects on intima-media thickness: an evaluation of rosuvastatin in Chinese subjects with subclinical atherosclerosis—design, rationale, and methodology of the METEOR-China study

**DOI:** 10.1186/s13063-020-04741-0

**Published:** 2020-11-11

**Authors:** Yilong Wang, Anxin Wang, Hongwei Li, Zhanquan Li, Bo Hu, Xiaogang Li, Huaguang Zheng, Lu Fu, Hongtao Hu, Zhiyu Nie, Yulin Qin, Bilian Zhao, Di Wei, Björn W. Karlson, Michiel L. Bots, Yundai Chen, Yongjun Wang

**Affiliations:** 1 Beijing Tian Tan Hospital, Capital Medical University, Beijing, China; 2 China National Clinical Research Center for Neurological Diseases, Beijing Tiantan Hospital, Capital Medical University, No. 6 Tiantanxili, Dongcheng District, Beijing, 100050 China; 3 Department of Cardiology, Beijing Friendship Hospital Affiliated to Capital Medical University, Beijing, China; 4 The People’s Hospital of Liaoning Province, Shenyang, China; 5 Department of Neurology, Union Hospital of Huazhong University of Science and Technology, Wuhan, China; 6 Department of Neurology, Peking University Third Hospital, Beijing, China; 7 The First Affiliated Hospital of Harbin Medical University, Harbin, China; 8 Beijing Ji Shui Tan Hospital, Beijing, China; 9 Department of Neurology, Shanghai Tongji Hospital, Tongji University School of Medicine, Shanghai, China; 10 AstraZeneca R&D, Shanghai, China; 11 AstraZeneca R&D Gothenburg, Mölndal, Sweden; 12 Department of Molecular and Clinical Medicine, Institution of Medicine, Sahlgrenska Academy, University of Gothenburg, Gothenburg, Sweden; 13 Julius Center for Health Sciences and Primary Care, University Medical Center Utrecht, Utrecht University, Utrecht, The Netherlands; 14 Department of Cardiology, China PLA General Hospital, 28 Fuxing Rd., Haidian District, Beijing, 100853 China

**Keywords:** Chinese, METEOR-China, Study design, Subclinical atherosclerosis, Rosuvastatin, Statin, Carotid intima-media thickness

## Abstract

**Background:**

The beneficial effect of statins on atherosclerosis and cardiovascular outcomes has been well established. The Measuring Effects on intima media Thickness: an Evaluation Of Rosuvastatin (METEOR) global study demonstrated that a 2-year orally administered treatment with rosuvastatin 40 mg daily significantly slowed the progression of carotid intima-media thickness (CIMT) compared to placebo. The current METEOR-China study is designed to evaluate the effect of rosuvastatin 20 mg daily versus placebo on the progression of atherosclerosis measured by CIMT in asymptomatic Chinese subjects.

**Methods:**

This is a phase 3, randomised, double-blind, placebo-controlled, multicentre parallel-group study. Asymptomatic Chinese subjects with a 10-year ischaemic cardiovascular disease (ICVD) risk < 10% will be recruited at 25 study sites. They will be treated with rosuvastatin 20 mg or placebo for 104 weeks. The primary endpoint is the annualised rate of change in CIMT measured by B-mode ultrasonography. Secondary endpoints include the annualised rate of change in CIMT at three different sections of the carotid artery and changes in the serum lipid profile. Safety parameters will also be assessed.

**Conclusion:**

The study will evaluate whether rosuvastatin 20 mg slows the progression of CIMT in asymptomatic Chinese subjects at low risk of ICVD.

**Trial registration:**

ClinicalTrials.gov NCT02546323. Registered on September 10, 2015

## Background

During the last two decades, the mortality and morbidity caused by cardiovascular disease (CVD) have increased significantly in China [1]. Atherosclerosis is the most important cause of cardiovascular and cerebrovascular diseases. Furthermore, atherosclerosis may have a silent course for decades before symptoms and atherothrombotic complications occur [2], by which time the disease already has major histopathological consequences that are poorly reversible. Therefore, early treatment based on the risk factors and signs of subclinical atherosclerosis is necessary and important. According to a national cross-sectional study involving 107,095 residents aged ≥ 40 years from the China National Stroke Prevention Project, for those 84,880 participants included in the analysis, the standardised prevalence of carotid atherosclerosis was 36.2%, showing the potential clinical importance of focusing on primary prevention of atherosclerosis progression [3].

Low-density lipoprotein cholesterol (LDL-C) is a major risk factor for atherosclerosis [4, 5]. Among lipid-lowering drugs, 3-hydroxy-3-methylglutaryl coenzyme A (HMG-CoA) reductase inhibitors (statins) are the cornerstone of therapy [6], and there is extensive evidence on the beneficial effects of statins on modification of the lipid profile, halting the progression of carotid and coronary atherosclerosis, and reduction of clinical cardiovascular morbidity and mortality [7–14]. Rosuvastatin is effective in lowering LDL-C and, like other statins, has favourable effects on other components of the lipid and lipoprotein profile, such as raising high-density lipoprotein cholesterol (HDL-C) and reducing levels of total cholesterol and triglycerides [15]. The principal evidence supporting the anti-atherosclerotic effects of rosuvastatin is based on the findings of the METEOR (Measuring Effects on intima media Thickness: an Evaluation Of Rosuvastatin; NCT00225589) study, the pivotal global phase 3 registration study that evaluated the effects of 2 years of treatment with rosuvastatin 40 mg on the natural history of atherosclerosis of the carotid arteries in 984 patients from 61 centres in eight countries [16]. Patients enrolled in METEOR were at low risk (defined as 10-year Framingham Risk Index < 10%) for coronary heart disease (CHD) according to the National Cholesterol Education Panel Adult Treatment Panel III (NCEP ATP III) 2001 guidelines and had subclinical atherosclerosis as assessed by carotid intima-media thickness (CIMT). Over the 2-year course of the study, rosuvastatin significantly slowed the progression of CIMT as compared with placebo. CIMT is considered a reliable surrogate marker for atherosclerosis [17], and using CIMT measurement as a marker for atherosclerosis may facilitate drug efficacy evaluation [18]. The major advantage of CIMT is that it is completely non-invasive and can be repeated as often as required.

Other small studies have shown the ability of rosuvastatin to slow the progression of atherosclerosis in Chinese and other Asian subjects. The REACH (Rosuvastatin Evaluation of Atherosclerotic Chinese Subjects) study showed that rosuvastatin may induce a rapid and lasting decrease in carotid plaque lipid content as assessed by magnetic resonance imaging [19]. In addition, the COSMOS (Coronary Atherosclerosis Study Measuring Effects of Rosuvastatin Using Intravascular Ultrasound in Japanese Subjects) study showed significant regression of coronary artery atheroma volume (assessed by intravascular ultrasound) in a Japanese patient population after a 76-week rosuvastatin treatment period [20].

The JUPITER (Justification for the Use of Statins in Prevention: an Intervention Trial Evaluating Rosuvastatin) outcome trial showed a significant 44% reduction in cardiovascular outcomes, including the reduction in mortality. JUPITER also showed that myopathy, hepatic injury, and cancer did not occur more frequently with rosuvastatin 20 mg than with placebo during its median follow-up of approximately 2 years of 17,802 patients from 26 countries [21].

However, most of the evidence of the effects of rosuvastatin comes from studies conducted out of China. Race/ethnicity may impact the magnitude of the association between the risk factors and the presence of atherosclerotic disease [22]. Since Asian individuals are generally smaller in terms of height and body surface area than Caucasian individuals, the dosage of lipid-modifying drugs may be different in the two populations. Given these observations, the Chinese food and drug regulatory agency required evidence on the efficacy and safety obtained from studies in native Chinese populations.

**Fig. 1:**
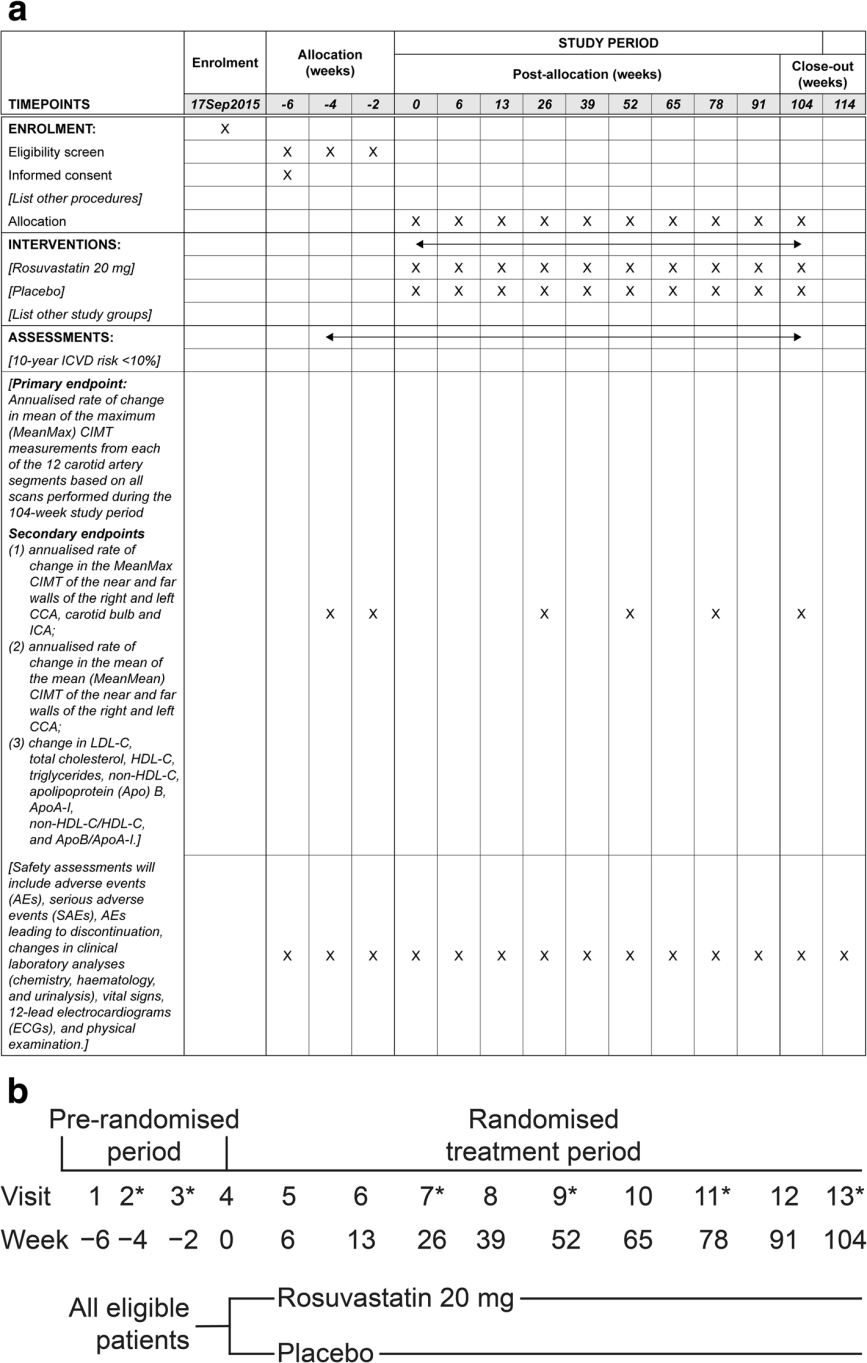
**a** Schedule of activities. **b** Study flow chart. *Intima-media thickness measurement will be performed at visits 2, 3, 7, 9, 11, and 13. *AE*, adverse event; *Apo*, apolipoprotein; *CCA*, common carotid artery; *CIMT*, carotid intima-media thickness; *ECG*, electrocardiogram; *HDL-C*, high-density lipoprotein cholesterol; *ICA*, internal carotid artery; *ICVD*, ischaemic cardiovascular disease; *LDL-C*, low-density lipoprotein cholesterol; *MeanMax*, mean of the maximum; *MeanMean*, mean of the mean; *SAE*, serious adverse event

We set out to conduct METEOR-China (ClinicalTrials.gov Identifier: NCT02546323) closely following the lessons learned from the global METEOR study. The principal objective of the METEOR-China study is to evaluate the effect of rosuvastatin (20 mg/day) versus placebo on the progression of CIMT in asymptomatic Chinese subjects with subclinical atherosclerosis who have a 10-year ischaemic cardiovascular disease (ICVD) risk < 10%. The design of this study is mainly based on that of the global METEOR study but includes only Chinese subjects, thereby complying with Chinese regulatory requirements by generating data from Chinese subjects (Schedule of activities [Fig. 1a] and SPIRIT checklist [supplementary Table 1] are included).

## Methods

### Study design

This is a phase 3, randomised, double-blind, placebo-controlled, multicentre, parallel-group study assessing the effects of rosuvastatin 20 mg treatment for 104 weeks on the change in CIMT in adult Chinese subjects with subclinical atherosclerosis. It consists of 13 study visits: three screening visits, one baseline visit, and nine treatment visits (Fig. 1b). There will be 25 centres across China.

### Study population

The study is designed to recruit asymptomatic Chinese subjects who have a 10-year ICVD risk < 10%. The ICVD risk is estimated based on the system of risk assessment introduced by the 2007 China Adult Dyslipidaemia Management Guidelines, in consideration of the Chinese ICVD prevalence characteristics that stroke is twice as prevalent as CHD [23]. According to this risk stratification system, the 10-year ICVD risk is equivalent to < 10% when LDL-C is ≥ 120 mg/dL (3.1 mmol/L) and < 190 mg/dL (4.9 mmol/L) for subjects without hypertension who have fewer than three other risk factors (including age), or when fasting LDL-C is ≥ 120 mg/dL (3.1 mmol/L) and < 160 g/dL (4.1 mmol/L) for subjects with only hypertension and age as ICVD risk factors, and subjects without hypertension who have three or more other risk factors (including age). Subjects need to have a maximum IMT of ≥ 1.2 mm and < 3.5 mm at any location in the carotid ultrasound scans. Detailed inclusion and exclusion criteria for the study are summarised in Table 1.

**Table 1:** Detailed inclusion and exclusion criteria for METEOR-China

**Inclusion criteria**	
• Provision of informed consent prior to any study-specific procedures	
• Male aged ≥ 45 and < 70 years or female aged ≥ 55 and < 70 years	
• Subjects with only hypertension (defined as blood pressure ≥ 140/90 mmHg or on antihypertensive treatment) and age (male ≥ 45 years, females ≥ 45 years) as CVD risk factors and subjects without hypertension who have three or more other risk factors (including age) must have the following: - Fasting LDL-C of ≥ 120 mg/dL (3.1 mmol/L) and < 160 mg/dL (4.1 mmol/L)	
• Subjects without hypertension who have fewer than three other risk factors (including age) must have the following: - Fasting LDL-C of ≥ 120 mg/dL (3.1 mmol/L) and < 190 mg/dL (4.9 mmol/L)	
• Triglycerides < 500 mg/dL (5.65 mmol/L) at visit 1 (week − 6)	
• HDL-C levels ≤ 60 mg/dL (1.6 mmol/L) at visit 1 (week − 6)	
• Maximum IMT ≥ 1.2 mm and < 3.5 mm at any location in the carotid ultrasound scans conducted at both visit 2 (week − 4) and visit 3 (week − 2)	
• Willing to follow all study procedures including study visits, fasting blood draws, and compliance with the study treatment regimen	
**Exclusion criteria**	
• Use of pharmacologic lipid-lowering medications (e.g. statins, fibrate derivatives, bile acid-binding resins, niacin, or its analogues at doses > 400 mg or prescribed Chinese traditional drugs), including CAIs and CAI/statin combination, within 12 months prior to visit 1 (week − 6)	
• Current or recent (within 2 weeks of visit 1, week − 6) use of supplements known to alter lipid metabolism (e.g. soluble fibres [including > 2 teaspoons Metamucil® or psyllium-containing supplement per day] or other dietary fibre supplements, marine oils, sterol/stanol products, or other supplement determined at the discretion of the investigator)	
• History of hypersensitivity reactions to other HMG-CoA reductase inhibitors	
• Pregnant women, women who are breastfeeding, and women of childbearing potential who are not using chemical or mechanical contraception or who have a positive serum pregnancy test	
• Clinical evidence of CAD or any other atherosclerotic disease such as angina, MI, transient ischaemic attack, symptomatic CAD, cerebrovascular accident, percutaneous coronary intervention, coronary artery bypass graft, peripheral arterial disease, and abdominal aortic aneurysm	
• History of cancer (other than basal cell carcinoma) in the past 2 years	
• Uncontrolled hypertension defined as either a mean resting diastolic blood pressure of ≥ 110 mmHg or a resting systolic blood pressure of ≥ 180 mmHg recorded at any time during the screening period	
• History of diabetes mellitus or current diabetes mellitus	
• Uncontrolled hypothyroidism defined as a thyroid-stimulating hormone > 1.5 times the ULN at visit 1 or subjects whose thyroid replacement therapy was initiated within the last 3 months	
• History of heterozygous or homozygous familial hypercholesterolaemia or known hyperlipoproteinaemia types I, III, IV, or V (familial dysbetalipoproteinaemia)	
• Use of the disallowed concomitant medications within 12 months prior to visit 1 (week − 6)	
• History of alcohol and/or drug abuse within the past 5 years	
• Active liver disease or hepatic dysfunction as defined by elevations of ≥ 1.5× ULN at visit 1 (week − 6) in any of the following liver function tests: ALT, AST, or bilirubin	
• Serum CK > 3× ULN at visit 1 (week − 6)	
• Serum creatinine > 2.0 mg/dL (177 mmol/L) recorded during the screening period	
• Participation in another investigational drug study and having ingested investigational drug ≤ 4 weeks before enrolment in the screening period	
• Previous randomisation in the present study	
• History of a significant medical or psychological condition that, in the opinion of the investigator, would compromise the subject’s safety or successful participation in the study	
• Involvement in the planning and/or conduct of the study (applies to both AstraZeneca staff and/or staff at the study site)	

*ALT* alanine aminotransferase, *AST* aspartate aminotransferase, *CAD* coronary artery disease, *CAI* cholesterol absorption inhibitor, *CK* creatine kinase, *CVD* cardiovascular disease, *HDL-C* high-density lipoprotein cholesterol, *HMG-CoA* 3-hydroxy-3-methylglutaryl coenzyme A, *IMT* intima-media thickness, *LDL-C* low-density lipoprotein cholesterol, *MI* myocardial infarction, *ULN* upper limit of normal

### Investigational products

The investigational product will be rosuvastatin 20 mg and placebo in oral tablet form for once daily use. The dose of 20 mg rosuvastatin has been selected and agreed with the regulatory authority as it is the maximum approved rosuvastatin dose in China. Rosuvastatin exposure is approximately 2-fold higher in Asian populations than in Western populations at the same dose [24]. Thus, it is expected that the 20 mg dose in Chinese people will yield similar exposure as 40 mg in Westerners.

A placebo control has been chosen so that the normal change in IMT can be ascertained for subjects who meet the study entry criteria. These criteria were selected to provide a population of subjects whose low risk of cardiovascular events warrant therapeutic lifestyle changes according to the 2007 China Adult Dyslipidaemia Management Guidelines [23].

Rosuvastatin 20 mg and the matching placebo will be prepared by the sponsor AstraZeneca and supplied in subject-specific labelled bottles. The randomisation schedule and treatment code will be assigned by an Interactive Voice Response System/Interactive Web Response System (IVRS/IWRS), and the site staff will dispense the investigational products to the study subjects accordingly.

### Modifications of the investigational products

Subjects may be discontinued from the investigational product in the following situations: creatine kinase > 10× upper limit of normal (ULN) accompanied by muscle pain, tenderness, or weakness; alanine aminotransferase (ALT) or aspartate aminotransferase (AST) > 3× ULN; deterioration in the subject’s condition that in the opinion of the investigator warrants subject withdrawal; and development of a condition that makes the subject high-risk, such as clinical atherosclerotic disease (myocardial infarction, transient ischaemic attack, stroke, angina pectoris, symptomatic coronary artery disease, peripheral arterial disease, abdominal aortic aneurysm) or diabetes mellitus. Subjects will be monitored to ensure that they are within the entry criteria limits for LDL-C throughout the study. If on two consecutive visits during the study LDL-C level exceeds the predefined range, the subject will be discontinued from the treatment.

### Concomitant medication

Other medications considered necessary for the subject’s safety and well-being may be given at the discretion of the investigator, provided that these medications do not conflict with the exclusion criteria. With the exception of lipid-regulating drugs, the only other disallowed drugs will be potent immunosuppressants, since the use of these agents may increase the risk of adverse events (AEs) during treatment with rosuvastatin.

### Efficacy endpoints

**Fig. 2:**
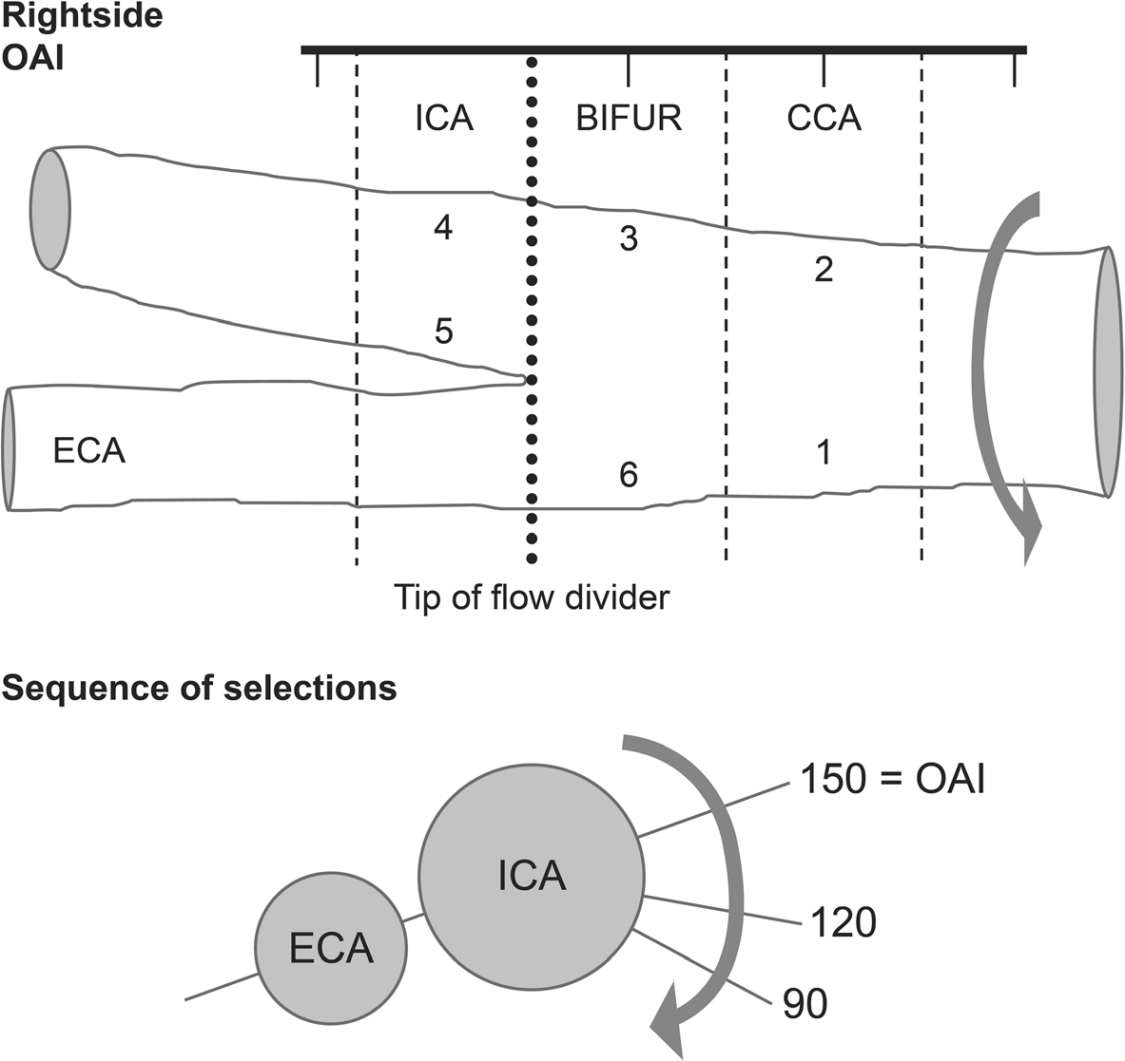
Schematic of the left carotid artery in the longitudinal section. BIFUR, bifurcation; CCA, common carotid artery; ECA, external carotid artery; ICA, internal carotid artery; OAI, optimal anatomical interrogation angle

CIMT assessment will be conducted at 12 carotid arterial segments: the near and far walls of the common carotid artery (CCA), the carotid bulb, and the internal carotid artery (ICA) of the right and left carotid arteries (Fig. 2).

The primary endpoint is the annualised rate of change in the mean of the maximum (MeanMax) CIMT measurements from each of the 12 carotid artery segments based on all scans performed during the 104-week study period. Secondary endpoints include the following: (1) annualised rate of change in the MeanMax CIMT of the near and far walls of the right and left CCA, carotid bulb, and ICA; (2) annualised rate of change in the mean of the mean (MeanMean) CIMT of the near and far walls of the right and left CCA; and (3) change in LDL-C, total cholesterol, HDL-C, triglycerides, non-HDL-C, apolipoprotein (Apo) B, ApoA-I, non-HDL-C/HDL-C, and ApoB/ApoA-I.

### Safety endpoints

Safety assessments will include AEs, serious adverse events (SAEs), AEs leading to discontinuation, changes in clinical laboratory analyses (chemistry, haematology, and urinalysis), vital signs, 12-lead electrocardiograms (ECGs), and physical examination. All AEs, including SAEs, will be collected from the time of signature of the informed consent throughout the treatment period and within 10 days after the last dose of the study drug. ECGs will be taken at visits 4 and 13. Laboratory safety assessments, vital signs, medical history, and physical examination will be performed to detect any sudden change in the condition according to the study plan shown in Table 2.

**Table 2:** Efficacy and safety parameter assessment schedule

Study plan	Screening			Treatment									
Visit number	1	2	3	4	5	6	7	8	9	10	11	12	13/ET^**a**^
Week number*	− 6	− 4	− 2	0	6	13	26	39	52	65	78	91	104
Informed consent	✓												
Randomisation				✓									
Vital signs	✓			✓	✓	✓		✓		✓		✓	✓
Height	✓												
Body weight	✓			✓									✓
Adverse events				✓	✓	✓	✓	✓	✓	✓	✓	✓	✓
Concomitant medications	✓			✓	✓	✓	✓	✓	✓	✓	✓	✓	✓
Medical history	✓												
Physical examination				✓									✓
ECG				✓									✓
Chemistry panel	✓^b^			✓	✓	✓		✓^c^		✓^c^		✓^c^	✓
Pregnancy test^d^	✓												
Haematology				✓									✓
Urine sample^e^				✓									✓
Serum lipid profile	✓			✓	✓	✓		✓		✓		✓	✓
ApoA-I and ApoB				✓									✓
Risk assessment	✓												
IMT		✓^f^	✓^f^				✓		✓		✓		✓✓^g^
Dispense investigational product				✓		✓	✓	✓	✓	✓	✓	✓	
Investigational product compliance					✓	✓	✓	✓	✓	✓	✓	✓	✓
TLC counselling^h^	✓			✓	✓	✓	✓	✓	✓	✓	✓	✓	

*ALP* alkaline phosphatase, *ALT* alanine aminotransferase, *Apo* apolipoprotein, *AST* aspartate aminotransferase, *CK* creatine kinase, *ECG* electrocardiogram, *ET* early termination, *IMT* intima-media thickness, *TLC* therapeutic lifestyle changes, *TSH* thyroid-stimulating hormone

*The visit window for all study visits is ± 7 days

^a^In the event of early termination, all non-IMT procedures scheduled for visit 13 (week 104) are to be conducted. A single IMT is to be performed on any subject who withdraws after 26 weeks

^b^Including TSH at visit 1

^c^Abbreviated chemistry panel including liver function tests (ALT, AST, bilirubin, ALP), serum creatinine, and CK only

^d^Urine pregnancy test conducted at the study site. Required only for premenopausal women. Those with amenorrhoea for at least 1 year are exempt

^e^For complete urinalysis

^f^IMT measurements at visit 2 (week − 4) and visit 3 (week − 2) must meet the inclusion criteria of maximum IMT ≥ 1.2 mm and < 3.5 mm

^g^Final IMT procedures will be scheduled before discontinuation of study treatment. The second and final IMT procedures should occur at or before visit 13 (week 104), at the time of discontinuation of the study treatment. The two IMT procedures for visit 13 should be performed on different days when possible

^h^TLC counselling is to be reinforced at each clinic visit

### CIMT measurements

CIMT assessment will be conducted at visits 2, 3, 7, 9, 11, and 13. Carotid ultrasound images are recorded digitally by sonographers, and then sent to the Ward A. Riley Ultrasound Center, Wake Forest School of Medicine, Winston-Salem, USA, or Vascular Imaging Center of the UMC Utrecht, Utrecht, The Netherlands, for centralised evaluation. To ensure accuracy and consistency of the measurements between the study centres, all sonographers and readers will participate in a uniform training and certification programme before the start of the study. The design of the trial with duplicate baseline and duplicate end of study measurements provide for continuous availability of reproducibility information during the conduct of the trial for quality assurance and quality control purposes.

High-quality standardised longitudinal B-mode images will be obtained at 12 well-defined arterial wall segments in both the right and left carotid arteries. Each carotid artery has been divided into three segments (CCA, carotid bulb, and ICA) with six walls (near wall and far wall of each segment) as shown in Fig. 1. The three segments are defined as follows: the near wall and far wall of the arterial segment extending from 10 to 20 mm proximal to the tip of the flow divider into the CCA; the carotid bifurcation beginning at the tip of the flow divider and extending 10 mm proximal to the flow divider tip; and the 10 mm of the ICA distal to the tip of the flow divider.

The ultrasound examination will start with an initial exploratory transverse/longitudinal scan, followed by a detailed longitudinal examination of the specific arterial segments illustrated in Fig. 1. The exploratory scans are to help the sonographer become familiar with the participant’s anatomy. During the transverse exploratory scan, the optimal anatomical interrogation (OAI) angle which best displays the tip of the flow divider and the “Y” appearance of the two arteries in a single longitudinal image can be determined. Based on the OAI angle with the tip of the flow divider positioned on the designated gridline, the sonographer can acquire a longitudinal exploratory image. Then, a detailed longitudinal scan for 12 segments at standardised angles can be performed. The images for each segment are selected at three predefined angles with 30° increments on both sides: 90, 120, and 150 for the right side and 210, 240, and 270 for the left side. The images obtained will be stored as digital media or uploaded via the internet for offline measurement of CIMT.

The CIMT is determined as the distance from the interface between the vessel lumen and the intima to the interface between the media and the adventitia. The primary endpoint for the study is change in the average maximum CIMT over 12 carotid segments. We refer to this parameter as the MeanMax CIMT. For each segment, the maximum CIMT value is obtained by combining data across the three interrogation angles and selecting the largest CIMT measurement. Then, the estimate of MeanMax CIMT is computed by averaging the maximum CIMT values for the 12 carotid segments. For one of the secondary endpoints, the value of MeanMean CCA will be evaluated by averaging the mean CIMT values across the three angles of interrogation [16].

### Laboratory measurements

Blood samples will be taken at the specified visits for analysis for serum lipids, haematology, pregnancy test, thyroid-stimulating hormone, and clinical chemistry (such as serum creatinine, liver function tests, creatine kinase). Subjects will have to fast for at least 8 h and will be seated for at least 5 min before blood samples are taken. Urine samples will be taken at specified visits.

### Statistical analysis

#### Sample size

With 207 subjects per group, there will be 90% power to detect a difference of − 10.6 μm/year in the change in the MeanMax CIMT of 12 vessel segments over a 104-week study period at a standard deviation of 33.28 μm/year and a 0.05 two-sided significance level. Adjusting for an 18% dropout rate, based on the global METEOR study, a total of approximately 506 subjects are to be randomised.

In the METEOR study, the difference in the annualised rate of change between the rosuvastatin and the placebo group was − 14.5 μm/year [16]. The effect size was downregulated 10% to − 13.1 μm/year, for a dose difference of 20 mg rosuvastatin in the current study versus 40 mg in the METEOR study. The 10% downregulation in CIMT was selected based on the relative effect of 20 mg versus 40 mg in LDL-C lowering in the VOYAGER meta-study [25]. The effect size was downregulated further 19% to − 10.6 μm/year because of the ethnic difference of Chinese subjects in the current study versus Caucasian subjects in the METEOR study. The 19% downregulation was selected based on the results from the MESA observational study [26] of CIMT over 10 years in different ethnicities in the USA.

#### Randomisation and blinding

Participants will be randomised by study investigators via the IVRS/IWRS to rosuvastatin 20 mg or placebo. Randomisation will be stratified by ICVD risk (< 5% or 5 to < 10%). The randomisation scheme will be generated in blocks to ensure approximate balance (1:1) between the two treatment arms. The randomisation scheme will be produced by using the AstraZeneca global randomisation system, which incorporates a standard procedure for generating random numbers.

Subjects, investigators, study site personnel, sonographers, ultrasound image readers, and sponsor personnel involved with data review and analysis will remain blinded to the study treatment throughout the study. The study treatment will be blinded by providing rosuvastatin and matching placebo tablets that are indistinguishable from each other in appearance and that will be presented in the same packaging.

Medication will be labelled using a unique kit ID number, which is linked to the randomisation scheme. The investigators, via the IVRS/IWRS, will allocate randomisation numbers sequentially when sites call the IVRS/IWRS to randomise an eligible subject. The IVRS/IWRS will also allocate the kit ID number to be dispensed to the subject.

AstraZeneca Patient Safety will have access in cases of emergency, and the drug supply chain will have access to enable supply of the investigational product. The central lab vendor will remain unblinded to the results of all laboratory tests. After screening, subjects and investigational site personnel will be blinded to blood lipid levels.

#### Efficacy

Three analysis sets will be used for data analysis: the intent-to-treat (ITT) population consists of all randomised subjects, the per-protocol (PP) population is a subset of the ITT population that includes subjects without any important protocol deviations, and the safety analysis set, which will consist of all subjects who take at least one dose of investigational product or placebo. The ITT population will be the primary efficacy analysis population. The CIMT analysis will be performed based on both the ITT and PP populations, and lipid and apolipoprotein analyses will be performed on the ITT population only. Safety parameters will be summarised on the safety analysis set.

A linear mixed effects (LME) model will be used for the primary analysis to assess the difference in the annualised rate of change in the CIMT measurements across 12 carotid artery sites between rosuvastatin 20 mg daily and placebo treatment over 104 weeks. For the primary efficacy analysis, the dependent variable is the maximum CIMT measurement from three interrogation angles at each of the 12 carotid artery sites at each time point of CIMT measurement during the 104-week study period. The model includes fixed effects for treatment group, time, time-by-treatment interaction, ICVD risk stratification (< 5% or 5 to < 10%), age, sex, and scan reader, and random effects for the intercept and slope at the individual subject level and carotid artery site within subject. Ultrasound machine will be included as a fixed effect as well, if different types of machines are deployed across sites. For the time of CIMT measurement, the two pre-randomisation visits and the final two visits at the end of the study will be treated as separate time points. Details will be defined in the statistical analysis plan. Time, as a continuous variable, is the interval from the date of randomisation to the date of CIMT measurement. Differences in the annualised change between rosuvastatin and placebo will be evaluated by testing the time-by-treatment interaction term. In the analysis, let *β*_1_ and *β*_2_ represent respectively the coefficient parameters for time and time-by-treatment interaction in the mixed effect model. The annualised rate of change (slope) in MeanMax CIMT in placebo will be estimated by the coefficient parameter *β*_1_, the annualised rate of change (slope) in MeanMax CIMT in rosuvastatin will be estimated by *β*_1_ + *β*_2_, and the difference of the annualised rate (slope) of change between the two groups will be estimated by *β*_2_. Then, the null and the alternative primary hypothesis can be expressed as *H*0: *β*_2_ = 0 versus Ha: *β*_2_ ≠ 0 at a significance level of 0.05 two sided. No imputation of missing CIMT measurements will be performed based on previous work indicating that the LME modelling was the most optimal approach, with no added value of imputation of missing data [27].

The same statistical method used for the primary CIMT efficacy analysis will be applied to model the segment-specific secondary CIMT efficacy outcome measurements. The difference in the annualised rate of change between rosuvastatin and placebo for the secondary CIMT outcome measures will also be evaluated by testing the time-by-treatment interaction term in each model fitting. Secondary lipid and lipoproteins outcome measures are the percentage change from baseline, which will be analysed by analysis of covariance with terms for treatment in the model. For lipid measurements (LDL-C, total cholesterol, HDL-C, triglycerides, non-HDL-C, and non-HDL-C/HDL-C), both the analysis of the per cent change from baseline at the final visit and the analysis of the per cent change from baseline to time-weighted average during treatment will be performed. In the evaluation of change from baseline to the final visit, missing observations will be imputed by the last observation carried forward (LOCF). The time-weighted average lipid value is defined as the lipid value multiplied by the number of days since the last lipid assessment, summed for all lipid observations and divided by the sum of days between all visits. No imputation will be made for missing lipid values for time-weighted average analysis. For apolipoprotein measurements (ApoB, ApoA-I, and ApoB/ApoA-I), the analysis of the per cent change from baseline to final visit (LOCF) will be performed. No multiplicity adjustment will be applied to the secondary efficacy analysis.

#### Safety

Safety outcome measures including incidence of AEs (including SAEs and AEs leading to discontinuation), changes in clinical laboratory analyses (chemistry, haematology, and urinalysis), vital signs, 12-lead ECGs, and physical examination will be summarised descriptively. Safety analysis will be conducted using the safety analysis set. No formal statistical test will be performed.

#### Data collection, management, and monitoring

The Rave Web Based Data Capture (WBDC) system will be used for data collection and query handling. Investigators will ensure that data will be recorded on the electronic case report form as specified in the study protocol and in accordance with the instructions provided.

All sonographers will attend group quality control meetings to review study data and to maintain a high degree of standardisation of the data collection process throughout the study.

The data collected through third-party sources will be obtained and reconciled with study data. Data queries will be raised for inconsistent, impossible, or missing data. All entries to the study database will be available in an audit trail. The data will be validated as defined in a data management plan. Quality control procedures will be applied to each stage of data handling to ensure that all data are reliable and have been processed correctly.

During the study, an AstraZeneca representative will have regular contacts with the study site to ensure data integrity and the quality of trial conduct. Throughout the study, the quality control centres will continuously monitor the quality of the images submitted from the routine studies and the outcome variable data obtained from the within- and between-sonographer and within- and between-reader quality control studies.

#### Ethics

An Independent Ethics Committee (IEC) will approve the final study protocol, including the final version of the informed consent form (ICF), and any other written information and/or materials that are provided to the subjects. The investigator will ensure the distribution of these documents to the applicable IEC and to the study site staff.

This study will be performed in accordance with the ethical principles that have their origin in the Declaration of Helsinki and that are consistent with the International Council for Harmonisation (ICH)/Good Clinical Practice (GCP), applicable Chinese regulatory requirements, and the AstraZeneca policy on Bioethics and Human Biological Samples. The principal investigator(s) at each site will ensure each subject is given full and adequate oral and written information about the study and that they are provided a signed and dated informed consent before conducting any study-related procedure. A copy of the signed ICF will be given to each subject. Any protocol amendment will be communicated to the investigators, ethics committees, and other relevant parties.

## Discussion

Several large, randomised, controlled studies have reported a beneficial effect of statin therapy on atherosclerosis [16, 28–33]. This effect of statins on carotid atherosclerosis was demonstrated using B-mode ultrasound measurement of CIMT, and on coronary atherosclerosis using quantitative coronary angiography and intravascular ultrasound. CIMT is commonly used [18], and has been recommended to assess overall CVD risk by the 2010 China Expert Consensus on Primary Prevention of CVD. The major advantage of CIMT is that it is completely non-invasive and can be repeated as often as required. It provides a continuous measure, since all subjects have a measurable carotid wall. It is also relatively inexpensive, and the technology is widely available [34]. Most important, CIMT is an accessible and reliable marker to assess subclinical atherosclerosis and cardiovascular risk [17]. For example, a meta-analysis of 119 randomised controlled trials involving 100,667 patients showed that the extent of interventions’ effects on CIMT progression predicted the degree of CVD risk reduction [35]. Furthermore, a CIMT trial may considerably improve the efficiency of the evaluation of new drug therapies on atherosclerosis and CVD risk. Hence, the results of a CIMT trial can be seen as a decision tool to support the development of drugs targeting atherosclerosis [36].

The METEOR-China study is a prospective, randomised study designed to assess the effects of long-term treatment with rosuvastatin on CIMT in “low CHD risk” Chinese subjects with subclinical atherosclerosis. The low risk has allowed for a placebo comparison, and the placebo control ascertains the normal change in IMT for Chinese subjects who meet the entry criteria.

The dose of 20 mg rosuvastatin has been selected for use in the current study as it is the maximum approved rosuvastatin dose in China (based on rosuvastatin exposure in Chinese patients). The intent is to arrest the progression of atherosclerosis to the same degree as was demonstrated in the METEOR trial, which used a dose of 40 mg rosuvastatin [16]. Also, the METEOR-China study affords the opportunity to assess the long-term safety of the 20-mg dose with a placebo comparator in a Chinese population.

In conclusion, given the continuously rising prevalence and mortality of CVD in China, primary prevention of atherosclerosis progression is becoming more and more urgent. The METEOR-China study will ascertain whether rosuvastatin 20 mg significantly slows the progression of subclinical atherosclerosis in addition to modifying serum lipid parameters, and assess the long-term safety profile of rosuvastatin 20 mg. The study has been designed to comply with the Chinese regulatory requirements by generating data from Chinese subjects, and thus bring new evidence and treatment options to reduce cardiovascular risk in Chinese people.

## Supplementary information

**Additional file 1.** SPIRIT Checklist: Recommended items to address in a clinical trial protocol and related documents.

## Data Availability

Following AstraZeneca’s transparency policy available at www.astrazenecaclinicaltrials.com

## Abbreviations

AE: Adverse event
ALT: Alanine aminotransferase
Apo: Apolipoprotein
AST: Aspartate aminotransferase
CCA: Common carotid artery
CHD: Coronary heart disease
CIMT: Carotid intima-media thickness
COSMOS: Coronary Atherosclerosis Study Measuring Effects of Rosuvastatin Using Intravascular Ultrasound in Japanese Subjects
CVD: Cardiovascular disease
ECG: Electrocardiogram
HDL-C: High-density lipoprotein cholesterol
HMG-CoA: 3-Hydroxy-3-methylglutaryl coenzyme A
ICA: Internal carotid artery
ICVD: Ischaemic cardiovascular disease
ITT: Intent-to-treat
IVRS: Interactive Voice Response System
IWRS: Interactive Web Response System
JUPITER: Justification for the Use of Statins in Prevention: an Intervention Trial Evaluating Rosuvastatin
LDL-C: Low-density lipoprotein cholesterol
LME: Linear mixed effects
LOCF: Last observation carried forward
METEOR: Measuring Effects on intima media Thickness: an Evaluation Of Rosuvastatin
NCEP ATP: National Cholesterol Education Panel Adult Treatment Panel
OAI: Optimal anatomical interrogation
PP: Per-protocol
REACH: Rosuvastatin Evaluation of Atherosclerotic Chinese Subjects
SAE: Serious adverse event
